# Development and Evaluation of an In-House Real-Time RT-PCR Targeting nsp10 Gene for SARS-CoV-2 Detection

**DOI:** 10.3390/ijms25063552

**Published:** 2024-03-21

**Authors:** Cyril Chik-Yan Yip, Jane Hau-Ching Poon, Kit-Hang Leung, Wan-Mui Chan, Jonathan Daniel Ip, Allen Wing-Ho Chu, Vincent Chi-Chung Cheng, Kwok-Yung Yuen, Kelvin Kai-Wang To

**Affiliations:** 1 Department of Microbiology, Queen Mary Hospital, Hong Kong SAR, China; vcccheng@hku.hk; 2 State Key Laboratory for Emerging Infectious Diseases, Carol Yu Centre for Infection, Department of Microbiology, School of Clinical Medicine, Li Ka Shing Faculty of Medicine, The University of Hong Kong, Pokfulam, Hong Kong SAR, China; janepn@hku.hk (J.H.-C.P.); kithang55@gmail.com (K.-H.L.); mbally@hku.hk (W.-M.C.); jdip1007@connect.hku.hk (J.D.I.); awhchu@hku.hk (A.W.-H.C.); kyyuen@hku.hk (K.-Y.Y.); kelvinto@hku.hk (K.K.-W.T.)

**Keywords:** SARS-CoV-2, COVID-19, nsp10, real-time RT-PCR, diagnostic

## Abstract

The emergence of SARS-CoV-2 mutations poses significant challenges to diagnostic tests, as these mutations can reduce the sensitivity of commonly used RT-PCR assays. Therefore, there is a need to design diagnostic assays with multiple targets to enhance sensitivity. In this study, we identified a novel diagnostic target, the nsp10 gene, using nanopore sequencing. Firstly, we determined the analytical sensitivity and specificity of our COVID-19-nsp10 assay. The COVID-19-nsp10 assay had a limit of detection of 74 copies/mL (95% confidence interval: 48–299 copies/mL) and did not show cross-reactivity with other respiratory viruses. Next, we determined the diagnostic performance of the COVID-19-nsp10 assay using 261 respiratory specimens, including 147 SARS-CoV-2-positive specimens belonging to the ancestral strain and Alpha, Beta, Gamma, Delta, Mu, Eta, Kappa, Theta and Omicron lineages. Using a LightMix E-gene RT-PCR assay as the reference method, the diagnostic sensitivity and specificity of the COVID-19-nsp10 assay were found to be 100%. The median Cp values for the LightMix E-gene RT-PCR and our COVID-19-nsp10 RT-PCR were 22.48 (range: 12.95–36.60) and 25.94 (range 16.37–36.87), respectively. The Cp values of the COVID-19-nsp10 RT-PCR assay correlated well with those of the LightMix E-gene RT-PCR assay (Spearman’s ρ = 0.968; *p* < 0.0001). In conclusion, nsp10 is a suitable target for a SARS-CoV-2 RT-PCR assay.

## 1. Introduction

In late December 2019, a new coronavirus, severe acute respiratory syndrome coronavirus 2 (SARS-CoV-2), was first detected in patients with pneumonia in Wuhan, China [1]. At that time, most patients infected with SARS-CoV-2 typically experienced symptoms such as fever, myalgia and cough and showed radiological findings of ground-glass lung opacities consistent with atypical pneumonia [2,3,4]. However, there have also been documented cases of asymptomatic or mild presentations [5,6,7,8,9,10].

SARS-CoV-2 is classified as a member of the genus *Betacoronavirus* in the family *Coronaviridae* [11]. It is an enveloped virus with a single-stranded positive-sense RNA genome of around 30 kilobase pairs in length. Its genome organization is similar to that of other coronaviruses, consisting of the following gene order: 5′- open reading frames 1a and 1b (ORF1a/1b), spike (S), envelope (E), membrane (M) and nucleocapsid (N)-3′. SARS-CoV-2 is highly contagious and can easily spread from person to person, leading to its rapid global dissemination [12,13,14,15,16]. This virus has caused the coronavirus disease 2019 (COVID-19) pandemic since 2020, resulting in significant global morbidity and mortality, with nearly 7 million deaths reported [17]. Following the relaxation of control measures, such as mask wearing and social distancing, the detection rates of common respiratory viruses, including SARS-CoV-2, have increased worldwide since 2023. RNA viruses, like SARS-CoV-2, are more prone to mutations compared to DNA viruses due to the lack of proofreading ability of their RNA-dependent RNA polymerase (RdRp) [18]. These factors will increase the likelihood of mutations in viral RNA genomes. Consequently, mismatches between virus genes and PCR primers/probes may occur frequently. As reported in various publications, mutations in primer or probe binding sites can impact the sensitivity of RT-PCR assays, leading to the occurrence of false-negative results [19,20,21,22]. To minimize such occurrences, it is crucial to design molecular diagnostic assays with multiple gene targets and search for a novel real-time RT-PCR target located in a conserved region of the genome. A timely and accurate diagnosis can significantly aid in facilitating appropriate antiviral treatment and implementing effective infection control measures.

In our previous studies, we successfully identified various abundantly expressed gene targets, including polymerase basic 2 (PB2) and nonstructural (NS) genes for influenza A virus, as well as nonstructural protein 1 (nsp1) and nsp8 genes for SARS-CoV-2 detection, using nanopore sequencing [23,24,25]. The diagnostic performance of these assays was comparable to that of other validated in-house or commercial RT-PCR assays. In the present study, we identified a novel gene target, nsp10, using the same strategy for the development of a highly sensitive and specific RT-PCR assay for COVID-19 diagnosis. In contrast to our previous gene targets (nsp1 and nsp8) of SARS-CoV-2, nsp10 was found to be highly conserved when compared to other genes. A study published in 2023 examined the prevalence of the most common mutation in each gene within the SARS-CoV-2 genome. The findings revealed that the prevalence of the most common mutation in nsp10 was found to be 0.16%, while the prevalence of the most common mutations in nsp1 and nsp8 was 8.01% and 0.7%, respectively [26]. Therefore, the conserved nature of the nsp10 gene reduces the likelihood of primer binding site mutations. We describe the evaluation of the in-house-developed COVID-19-nsp10 RT-PCR assay using clinical specimens and external quality assessment (EQA) samples.

## 2. Results

### 2.1. Design of a Novel COVID-19 Real-Time RT-PCR Assay Targeting the nsp10 Gene of SARS-CoV-2

**Figure 1:**
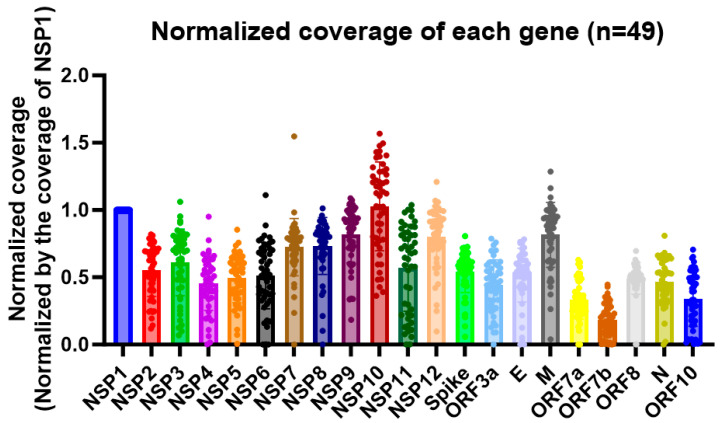
The normalized coverages of different genes in the SARS-CoV-2 genome from 49 whole-genome-sequenced clinical specimens. The coverage of each gene was calculated by averaging the number of reads mapped to each position of the same gene. The coverage of the NSP1 gene was used to normalize the coverages of the other genes and the desired region for comparison. The Y-axis represents the normalized coverage, and the X-axis shows the genes.

**Figure 2:**
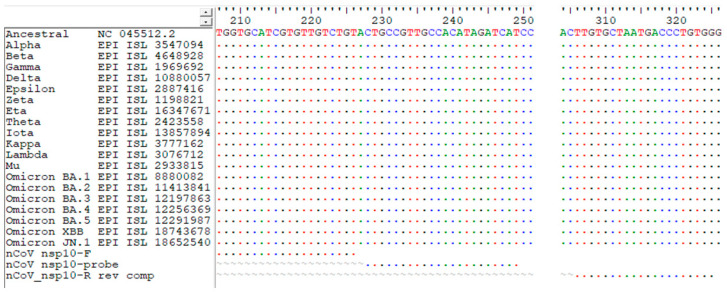
Multiple sequence alignment using our primer and probe sequences and the nsp10 gene sequences of SARS-CoV-2 variants from different geographical regions. The alignment on the left contains the alignment of the forward primer sequence and the probe sequence together with other reference sequences. The alignment on the right contains the alignment of the reverse complement of the reverse primer sequence with other reference sequences. The dots represent bases that are the same as the ones in the ancestral sequence (NC_045512.2). The ‘~’ (tilde) symbols in the figure represent gaps in the alignment. However, the gaps do not represent deletions in the sequence. A more detailed view of the alignment of the sequences can be referred to the Supplementary Materials (Supplementary Figure S1).

In this study, we observed a high sequencing coverage of the nsp10 gene of SARS-CoV-2 in 49 clinical samples, as demonstrated by nanopore whole-genome sequencing with the nanopore protocol for the PCR tiling of COVID-19 (PTC_9096_v109_revH_06Feb2020). Among all the genes in the SARS-CoV-2 genome, the nsp10 gene had the highest coverage (adjusted *p* value < 0.0001) (Figure 1). Also, despite the high sequencing coverage of nsp10 in the clinical specimens, the large variances in the normalized coverages highlighted the importance of having different diagnostic targets for RT-PCR assays (Figure 1). Out of the 49 patients, 29 of them (59.2%) had a higher coverage in nsp10 compared to in nsp1. The patient with the highest normalized nsp10 coverage had a coverage that was 56.8% higher than that of nsp1, while the patient with the lowest normalized nsp10 coverage had a coverage that was 63.7% lower than that of nsp1. As for nsp8, 42 out of 49 patients (85.7%) had a higher coverage in nsp10 compared to in nsp8. The patient with the largest difference in coverage between nsp10 and nsp8 had an nsp10 coverage that was 3.7 times higher than the nsp8 coverage, while one of the patients’ nsp8 gene could not be sequenced. Thus, we designed primers and a probe targeting the nsp10 gene as a new target for our in-house-developed RT-PCR assay (Table 1). Multiple sequence alignment demonstrated that the target sites of our nsp10 primers and probe were well conserved among different variants (the ancestral strain, Alpha, Beta, Gamma, Delta, Epsilon, Zeta, Eta, Theta, Iota, Kappa, Lambda, Mu and Omicron [including JN.1]) (Figure 2 and Figure S1).

### 2.2. Analytical Sensitivity and Specificity of the COVID-19-nsp10 Real-Time RT-PCR Assay

To evaluate the analytical sensitivity of the in-house-developed COVID-19-nsp10 RT-PCR assay, the limit of detection (LOD) was determined by using two-fold serial dilutions of a total nucleic acid (TNA) extracted from SARS-CoV-2 Q control 01 and tested in quadruplicate in two independent runs. The LOD of the COVID-19-nsp10 RT-PCR assay was determined to be 74 copies/mL (95%CI 48–299 copies/mL) by a probit analysis (Table 2).

To evaluate the analytical specificity of the COVID-19-nsp10 RT-PCR assay, we tested the TNA of human coronaviruses SARS-CoV-1, Middle East Respiratory Syndrome Coronavirus (MERS-CoV), HCoV-OC43, HCoV-HKU1, HCoV-229E and HCoV-NL63 and other common respiratory viruses, including rhinovirus, adenovirus, influenza A viruses (H1N1 pdm09 and H3N2), influenza B virus, influenza C virus, parainfluenza virus types 1–4, respiratory syncytial virus and human metapneumovirus. Our in-house-developed COVID-19-nsp10 RT-PCR assay showed no cross-reaction with these respiratory viruses.

### 2.3. Diagnostic Performance of the COVID-19-nsp10 Assay for SARS-CoV-2 Detection

**Figure 3:**
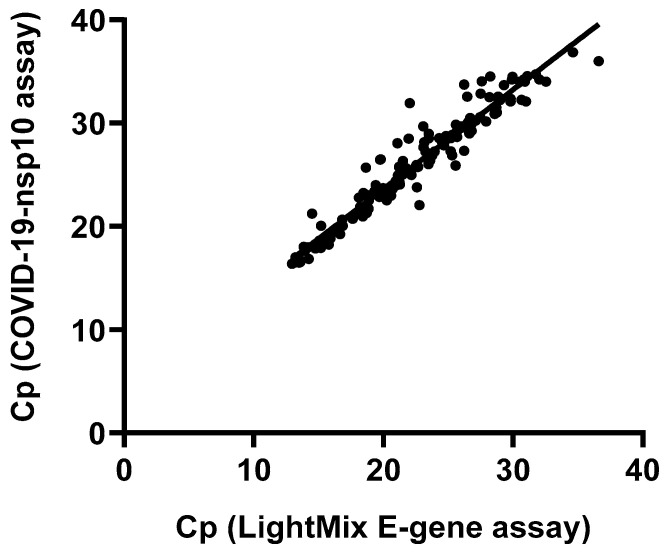
Correlation of the Cp values of the samples found positive for SARS-CoV-2 by the in-house COVID-19-nsp10 assay and the LightMix E-gene assay.

**Figure 4:**
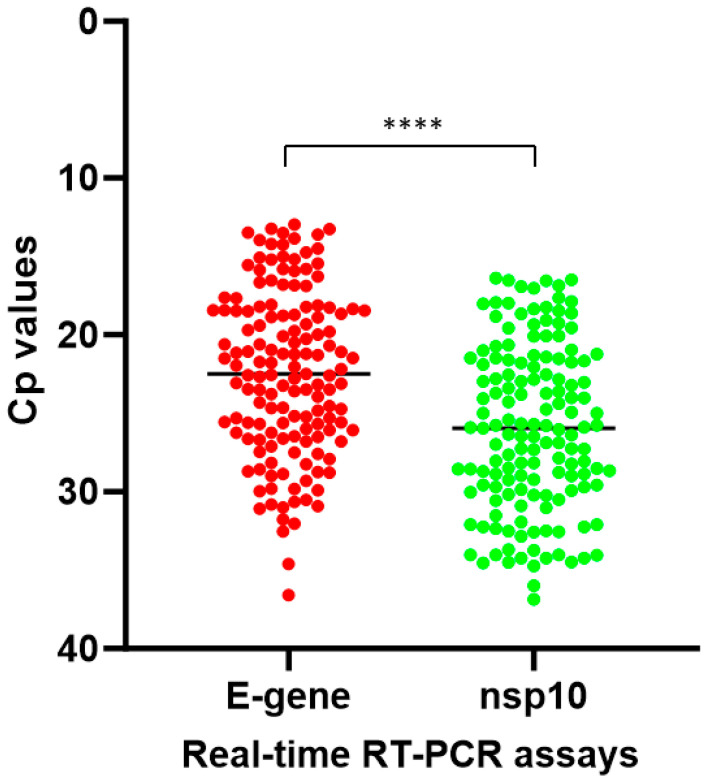
Comparison of the Cp values of the COVID-19-nsp10 and LightMix E-gene assays. **** indicates *p* < 0.0001.

To evaluate the diagnostic performance of our in-house-developed COVID-19-nsp10 real-time RT-PCR assay, 261 respiratory specimens collected from hospitalized patients suspected of having COVID-19 were analyzed for the presence of SARS-CoV-2. Among these 261 clinical specimens collected during 2020–2023, 147 (56.3%) tested positive for SARS-CoV-2 by both the in-house-developed COVID-19-nsp10 RT-PCR (Cp range: 16.37–36.87) and the commercial LightMix E-gene RT-PCR (Cp range: 12.95–36.60). A good agreement between the performance of the in-house COVID-19-nsp10 RT-PCR assay and that of the LightMix E-gene RT-PCR assay was revealed by a strong correlation (Spearman’s ρ = 0.968; *p* < 0.0001) (Figure 3). The median Cp value of the COVID-19-nsp10 RT-PCR assay (25.94) was significantly higher than that of the LightMix E-gene RT-PCR assay (22.48; *p* < 0.0001) (Figure 4). Using the commercial LightMix E-gene RT-PCR as the reference assay, the diagnostic sensitivity and specificity of the COVID-19-nsp10 RT-PCR assay were found to be 100% (Table 3). Nanopore sequencing was used to identify the lineages of SARS-CoV-2 present in the 147 positive specimens, and the sequencing results showed that the isolates belonged to a broad range of variants, including the ancestral strain and Alpha, Beta, Gamma, Delta, Mu, Eta, Kappa, Theta and Omicron variants. Among six EQA samples from the College of American Pathologists (CAP), both assays gave 100% correct results.

## 3. Discussion

In this study, we developed a real-time RT-PCR assay targeting the nsp10 gene of SARS-CoV-2 for COVID-19 diagnosis. Given the ongoing emergence of new variants with mutations scattered across the viral genome [27,28,29,30,31], it is advisable to design primers and probes that target different genes to ensure the accurate detection of these variants. In December 2023, the World Health Organization classified the recently emerged JN.1 as a variant of interest (VOI) following its rapid spread in multiple countries [32]. The identification of a newly emerged variant further highlights the need for robust diagnostic assays that can detect various SARS-CoV-2 strains. While the N gene of SARS-CoV-2 is commonly used as a target for COVID-19 nucleic acid amplification tests, instances of N gene target failure have been reported [33,34]. Hence, it is crucial to identify alternative conserved target genes for SARS-CoV-2 detection. The nsp10 gene was found to be abundantly covered by sequencing reads in clinical specimens (Figure 1) and conserved among different SARS-CoV-2 variants and different genes [26], further supporting its potential as an attractive target for SARS-CoV-2 detection. Different from the nsp1 and N genes, the nsp10 gene is located near the center of the genome in the ORF1ab region. During the life cycle of the SARS-CoV-2 virus, nsp10 is essential for replicating and proofreading viral RNA [35]. The conservation of nsp10 is likely due to its importance in the viral life cycle and the need to maintain its interaction with RdRp. Notably, a structural analysis by Wang et al. showed that nsp10 has limited room for conformational changes, which also contributes to the conserved nature of the nsp10 protein [36]. Variations in the nsp10 gene can have significant implications in SARS-CoV-2 viral evolution because changes in the nsp10 gene can be accompanied by changes in the fundamental function of the nsp10 protein and its role in viral replication. In terms of surveillance, differences in the nsp10 genes of different SARS-CoV-2 strains could signify a more distant phylogenetic relationship, which could be useful for the surveillance and monitoring of virus spread. In the present study, we also observed a high variability in the sequencing coverage of different genes within the SARS-CoV-2 genome. This finding reemphasizes the importance of having multiple targets for real-time RT-PCR assays. Also, the variance in the sequencing coverage of the nsp10 gene was high (Figure 1), given the high conservation of its genomic sequence. This can be attributed to various factors, such as viral load and sample quality.

Our COVID-19-nsp10 assay demonstrated high sensitivity and specificity in detecting all evaluated variants, including the recently emerged Omicron variants XBB and JN.1. The in-house-developed COVID-19-nsp10 assay exhibits high sensitivity, with an LOD below 100 copies/mL, and it does not show any cross-reactivity with other common respiratory viruses. The commercial LightMix E-gene assay is commonly used as a reference assay due to its exceptional sensitivity in detecting SARS-CoV-2 [37,38,39,40,41,42,43,44], with an LOD of 31.3 copies/mL, as determined in our previous study [25]. When compared to the validated LightMix E-gene assay as the reference method, our COVID-19-nsp10 assay showed comparable diagnostic performance, with 100% diagnostic sensitivity and specificity using clinical specimens. The median Cp value of the in-house-developed COVID-19-nsp10 assay was found to be significantly higher than that of the commercial LightMix E-gene assay. This difference in Cp value may be attributed to several factors, such as the lower volume of sample extract used in the in-house assay compared to in the E-gene assay, as well as primer and probe design, potential variations in PCR reagents and thermal cycling conditions between the two assays. However, despite this discrepancy, there was no significant difference in the diagnostic sensitivity between the two assays. In addition, the Cp values obtained from both the COVID-19-nsp10 and LightMix E-gene assays exhibited an excellent correlation. We further evaluated the performance of our COVID-19-nsp10 assay and the LightMix E-gene assay using EQA samples from the CAP. Both assays performed well, reaffirming their accuracy and reliability in detecting SARS-CoV-2. These findings indicate that our in-house COVID-19-nsp10 assay, along with the LightMix E-gene assay, demonstrates excellent diagnostic performance for the detection of SARS-CoV-2 RNA. When comparing the sensitivity of our in-house COVID-19-nsp10 assay with that of our previously developed COVID-19-RdRp/Hel and N assays, the COVID-19-nsp10 assay was found to be more sensitive than the latter two assays (>400 copies/mL) [45]. However, when we modified the COVID-19-RdRp/Hel and N assays to be in a single-tube nested format, the sensitivities of both nested assays improved by nearly one log_10_ [46]. Therefore, the sensitivity of a PCR assay is not solely determined by the gene target but can also be influenced by other factors, such as assay design.

Apart from analytical and diagnostic performance, there are other essential factors that need to be considered when selecting diagnostic assays, such as turnaround time (TAT) and cost, particularly in the context of a pandemic, where there is a high volume of clinical samples from patients suspected of having COVID-19. Regarding TAT, both the COVID-19-nsp10 and E-gene assays had the same sample-to-extract time since the same extraction method was utilized. Nevertheless, the E-gene assay had a shorter PCR running time (42 min) compared to our in-house COVID-19-nsp10 assay (72 min). In terms of cost, considering the expenses associated with PCR reagents and primers/probes, our in-house COVID-19-nsp10 assay (USD 2 per reaction) proved to be significantly more cost-effective compared to the commercial LightMix E-gene assay (USD 10 per reaction). For laboratories with expertise in molecular assay development, utilizing in-house-developed tests can result in substantial cost savings, particularly when managing a large number of samples during a pandemic.

There are some limitations in this study. Due to the limited number of clinical specimens for each patient, we were not able to classify each sample into groups of different sequencing coverage of different genes by sequencing and then compare the sensitivity of different RT-PCR assays. Therefore, an interesting direction for future projects would be to evaluate the sensitivity of different assays given different sequencing coverage of the genes. Also, using NGS to search for novel diagnostic targets in the virus genome can be risky, as researchers can be misled by the unrelated sequencing biases induced during the library preparation step. Therefore, verification of RT-PCR assays by comparing their performance with that of the current gold standard is recommended.

In conclusion, a low-cost, sensitive, specific and reliable real-time RT-PCR assay targeting the nsp10 gene of SARS-CoV-2 was successfully developed for COVID-19 diagnosis. Next-Generation Sequencing is a valuable tool for identifying suitable gene targets for molecular test development by analyzing the expression of each gene.

## 4. Materials and Methods

### 4.1. Viruses and Samples Used for Evaluation

Two-fold serial dilutions of viral nucleic acid extracts of SARS-CoV-2 Q control 01 (Qnostics, Glasgow, UK) were used for an analytical sensitivity evaluation. The viral nucleic acid extracts of a clinical sample containing human coronavirus HCoV-HKU1 and 17 culture isolates of other human coronaviruses (HCoV-OC43, HCoV-NL63, HCoV-229E, MERS-CoV and SARS-CoV-1) and respiratory viruses (rhinovirus, adenovirus, influenza A viruses (pdm09 H1N1 and H3), influenza B virus, influenza C virus, respiratory syncytial virus, human metapneumovirus and parainfluenza virus types 1–4) were used for an analytical specificity evaluation [45]. A total of 261 clinical samples, including nasopharyngeal aspirate, nasopharyngeal swab, throat swab or posterior oropharyngeal saliva samples, collected from hospitalized patients (male–female = 119:142; median age: 69 years; range: 3 months–106 years) suspected of having COVID-19 were included for a diagnostic performance evaluation. Among these 261 samples collected between 2020 and 2023, 17 were from the year 2020, 65 were from the year 2021, 16 were from the year 2022, and 163 were from the year 2023. The lineages of SARS-CoV-2 in the positive samples were identified by nanopore sequencing. Apart from these clinical samples, we also evaluated six EQA samples from the CAP in year 2023 that had various concentrations of SARS-CoV-2 or were negative for SARS-CoV-2.

### 4.2. Whole-Genome Sequencing of SARS-CoV-2

Whole-genome sequencing was performed using an Oxford Nanopore MinION device (Oxford Nanopore Technologies, Oxford, UK). Library preparation was performed following the Nanopore protocol—PCR tiling of COVID-19 virus (Version: PTC_9096_v109_revH_06Feb2020)—according to the manufacturer’s instructions with minor modifications (Oxford Nanopore Technologies), as we described previously [47]. Briefly, RNA was first extracted and reverse-transcribed to cDNA using SuperScript IV reverse transcriptase (Thermo Fisher Scientific, Waltham, MA, USA). cDNA was then amplified using hCoV-2019/nCoV-2019 Version 3 Amplicon Set or hCoV-2019/nCoV-2019 Version 4.1 Amplicon Set (Integrated DNA Technologies, Coralville, IA, USA) with a Q5^®^ Hot Start High-Fidelity 2X Master Mix kit (New England Biolabs, Ipswich, MA, USA). PCR products were then purified with 1x AMPure XP beads (Beckman Coulter, Brea, CA, USA) and quantified with a Qubit dsDNA HS Assay kit (Thermo Fisher Scientific). The purified PCR products were then normalized for end-repair and native barcode ligation reactions with Native Barcoding Expansion 96 (EXP-NBD196, Oxford Nanopore Technologies). Barcoded libraries were then pooled together, purified with 0.4× AMPure XP beads and quantified with a Qubit dsDNA HS Assay kit (Thermo Fisher Scientific). The purified pooled libraries were ligated to sequencing adapters and sequenced on the Oxford Nanopore MinION device using R9.4.1 flow cells for 24–48 h.

### 4.3. In Silico Analysis

The raw reads from the 49 sequenced clinical specimens were quality-checked. The sequencing primers were removed by trimming the extreme ends of each read. They were then mapped to the SARS-CoV-2 ancestral strain (GenBank accession number: NC_045512.2) using Medaka software v1.0.3 [48]. The BAM files of the corresponding specimens were extracted and indexed for further analysis. The pysamstats module was used to extract the coverage of each position of each specimen across the whole SARS-CoV-2 genome [49]. The coverage of each gene was calculated by taking the average coverage of the bases located in different genes. The nucleotide coordinates of each gene were extracted from the National Center for Biotechnology Information (NCBI). The coverage of each gene was then normalized by the coverage of the nsp1 gene. Then, the normalized coverage of each gene was exported as a CSV file. A bar chart was created using PRISM v10.0.2.

Primers and a probe targeting the nsp10 gene region of SARS-CoV-2 for the in-house-developed RT-PCR assay were designed and tested. Multiple sequence alignment was performed by the ClustalX version 2.1 program using our primer/probe sequences and the nsp10 gene sequences of SARS-CoV-2 variants from different countries or geographical regions [50]. The SARS-CoV-2 ancestral strain sequence (GenBank accession number: NC_045512.2) and the sequences of other SARS-CoV-2 variants were retrieved from the Global Initiative on Sharing All Influenza Data (GISAID) EpiCoV database or the NCBI GenBank database.

### 4.4. Nucleic Acid Extraction

A MagNA Pure 96 extraction system (Roche, Basel, Switzerland) was used for total nucleic acid extraction according to the manufacturer’s instructions, as previously described [25]. In brief, 200 μL of each sample was mixed with 250 μL of MagNA Pure External Lysis Buffer (Roche), which contains a chaotropic salt and a detergent to lyse cells and release nucleic acid. The lysed sample was then loaded onto the MagNA Pure 96 instrument, which automatically performs the extraction protocol, including binding, washing and elution steps. The extracted TNA was then recovered in 50 μL of elution buffer and kept at −80 °C until further use.

### 4.5. Real-Time RT-PCR Assays for SARS-CoV-2 RNA Detection

We used a QuantiNova Probe RT-PCR Kit (QIAGEN, Hilden, Germany) to perform the in-house-developed COVID-19-nsp10 real-time RT-PCR assay for SARS-CoV-2 detection. Each reaction mixture (20 μL) consisted of 10 μL of 2× QuantiNova Probe RT-PCR Master Mix, 0.2 μL of QN Probe RT-Mix, 1.6 μL of each 10 μM forward and reverse primer, 0.4 μL of 10 μM probe (Table 1), 1.2 μL of nuclease-free water and 5 μL of TNA. A LightCycler 480 II Real-Time PCR System (Roche) was used to perform the in-house RT-PCR. The thermal cycling conditions consisted of 45 °C for 10 min and 95 °C for 5 min, followed by 45 cycles of 95 °C for 5 s and 55 °C for 30 s. Positive and negative controls were included in each run to monitor RT-PCR performance.

We used the commercial LightMix E-gene kit for SARS-CoV-2 detection (TIB Molbiol, Berlin, Germany), along with LightCycler Multiplex RNA Virus Master (Roche), as the reference assay in this study. Each reaction mixture (20 μL) contained 4 μL of Roche RT-qPCR Reaction Mix, 0.5 μL of TIB Molbiol primer/probe reagent mix, 0.1 μL of Roche RT Enzyme Solution, 5.4 μL of nuclease-free water and 10 μL of TNA. The LightCycler 480 II real-time PCR system (Roche) was used to perform the E-gene assay. The thermal cycling conditions consisted of 55 °C for 3 min and 95 °C for 30 s, followed by 45 cycles of 95 °C for 3 s and 60 °C for 12 s [25,37]. Positive and negative controls were used in each run.

### 4.6. Statistical Analysis

Agreement between the in-house-developed RT-PCR assay and the reference RT-PCR assay was determined by the kappa statistic. The performance of the in-house RT-PCR assay was evaluated against that of the reference RT-PCR assay using McNemar’s test. Spearman’s correlation analysis was conducted to evaluate the relationship between the Cp values obtained from the two real-time RT-PCR assays performed in this study. The Cp values obtained from the two real-time RT-PCR assays were compared using Wilcoxon signed-rank test. *p* < 0.05 was considered statistically significant. Statistical analyses were conducted using either GraphPad Prism 10 or IBM SPSS Statistics 27 software.

## Figures and Tables

**Table 1:** Primers and probe used in this study.

Primer/Probe ^1^	Sequence (5′-3′)	Position ^2^
nCoV_nsp10-F	TGGTGCATCGTGTTGTCTGT	13,231–13,250
nCoV_nsp10-R	CCACAGGGTCATTAGCACAA	13,330–13,349
nCoV_nsp10-probe	FAM-CTGCCGTTGCCACATAGATCAT-IABkFQ	13,252–13,273

^1^ F, forward primer; R, reverse primer. ^2^ Primer and probe position corresponding to the genome sequence of SARS-CoV-2 isolate Wuhan-Hu-1 (GenBank: NC_045512.2).

**Table 2:** Test results for determining the limit of detection of the in-house-developed COVID-19-nsp10 RT-PCR assay with TNA extracted from Qnostics SARS-CoV-2 Q control 01.

Concentration (Copies/mL)	Cp (Intra-Run)				Cp (Inter-Run)			
	Test 1	Test 2	Test 3	Test 4	Test 1	Test 2	Test 3	Test 4
500	36.31	35.62	36.49	36.25	35.92	35.35	35.86	35.25
250	37.09	36.65	37.02	37.01	36.85	35.68	36.07	36.59
125	38.07	38.73	37.78	36.94	37.17	37.14	36.76	37.17
62.5	37.23	38.66	38.13	-	37.92	37.2	36.71	39.12
31.3	-	40	-	40	38.48	38.39	-	37.68
15.6	-	-	-	-	38.56	-	-	-
NTC	-	-	-	-	-	-	-	-

Cp, crossing point; NTC, no-template control; -, not detected.

**Table 3:** Diagnostic performance of the in-house-developed COVID-19-nsp10 assay compared to that of the LightMix E-gene RT-PCR assay for SARS-CoV-2 detection using clinical specimens.

Molecular Assay		LightMix E-Gene RT-PCR			
		Positive	Negative	Kappa Value (95% CI) ^1^	McNemar’s Test
**COVID-19-nsp10 real-time RT-PCR**	**Positive**	147	0	1.00 (1.00–1.00)	*p* = 1.000
	**Negative**	0	114		

^1^ CI, confidence interval.

## Data Availability

The data presented in this study are available on request from the corresponding author. The data are not publicly available due to privacy/ethical restrictions.

## Supplementary Materials

The following supporting information can be downloaded at: https://www.mdpi.com/article/10.3390/ijms25063552/s1.
