# Supplementary material for: Development and Evaluation of an In-House Real-Time RT-PCR Targeting nsp10 Gene for SARS-CoV-2 Detection
Source: Int J Mol Sci. 2024 Mar 21;25(6):3552. doi: 10.3390/ijms25063552 (PMC10970969; doi:10.3390/ijms25063552)
Supplement: Supplementary file 1 [file ijms-25-03552-s001.zip › Supplementary Figure S1.pdf]

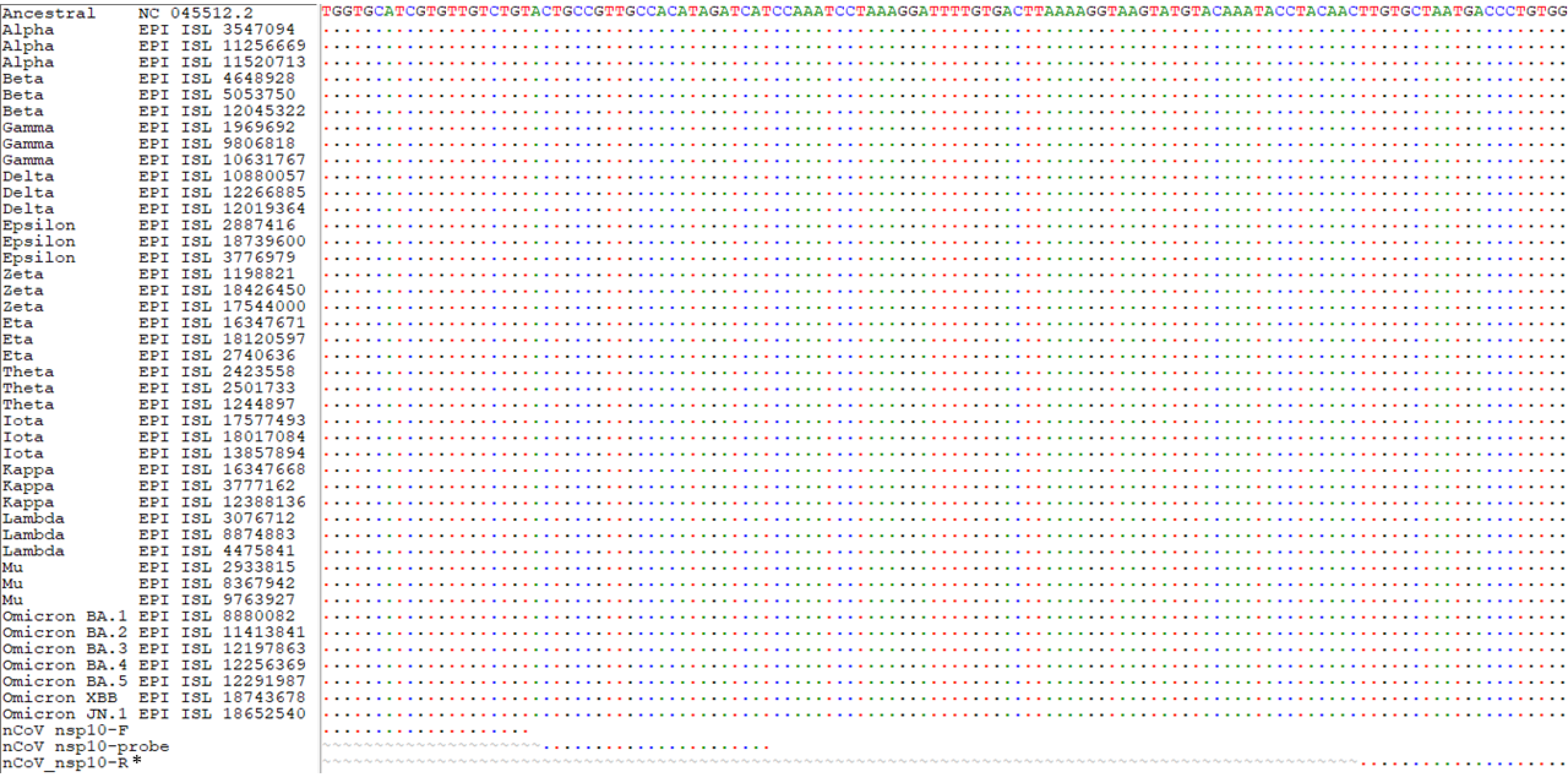

* Reverse complement

Figure legend of Figure S1

Multiple sequence alignment of our primer and probe sequences and the nsp10 gene sequences of SARS-CoV-2 variants from different geographical regions. The alignment contains the primer and probe sequences with other reference sequences. The dots represent bases which were the same as the one in the ancestral sequence (NC_045512.2). The '~' (tilde) symbols in the figure represent gaps in the alignment. However, the gaps do not represent deletions in the sequence.
