# Supplementary material for: Development and Evaluation of an In-House Real-Time RT-PCR Targeting nsp10 Gene for SARS-CoV-2 Detection
Source: Int J Mol Sci. 2024 Mar 21;25(6):3552. doi: 10.3390/ijms25063552 (PMC10970969; doi:10.3390/ijms25063552)
Supplement: Supplementary file 1 [file ijms-25-03552-s001.zip › Supplementary Table S1.pdf]

SUPPLEMENTAL TABLE

Data Availability

GISAID Identifier:	EPI_SET_240125wy
doi:	10.55876/gis8.240125wy

All genome sequences and associated metadata in this dataset are published in GISAID's EpiCoV database. To view the contributors of each individual sequence with details such as accession number, Virus name, Collection date, Originating Lab and Submitting Lab and the list of Authors, visit 10.55876/gis8.240125wy

Data Snapshot

- EPI_SET_240125wy is composed of 41 individual genome sequences.
- The collection dates range from 2021-01-24 to 2023-12-24;
- Data were collected in 12 countries and territories;
- All sequences in this dataset are compared relative to hCoV-19/Wuhan/WIV04/2019 (WIV04), the official reference sequence employed by GISAID (EPI_ISL_402124). Learn more at https://gisaid.org/WIV04.
