# Supplementary material for: Development and Evaluation of an In-House Real-Time RT-PCR Targeting nsp10 Gene for SARS-CoV-2 Detection
Source: Int J Mol Sci. 2024 Mar 21;25(6):3552. doi: 10.3390/ijms25063552 (PMC10970969; doi:10.3390/ijms25063552)
Supplement: Supplementary file 1 [file ijms-25-03552-s001.zip › Supplementary Table S2.pdf]

Supplementary Table S2. The Uniform Resource Locator (URL) links, their respective regular links, and their respective access dates shown in Supplementary Table S1.

Link description	Doi link	Regular link	Access date
Acknowledgement of Data Contributors	10.55876/gis8.240125wy	https://epicov.org/epi3/epi_set/240125wy?main=true	31 January 2024
Official hCoV-19 Reference Sequence	N/A	https://gisaid.org/WIV04/	31 January 2024
